# 5-Hydroxymethylcytosine signatures in circulating cell-free DNA as diagnostic and predictive biomarkers for coronary artery disease

**DOI:** 10.1186/s13148-020-0810-2

**Published:** 2020-01-21

**Authors:** Chaoran Dong, Jiemei Chen, Jilin Zheng, Yiming Liang, Tao Yu, Yupeng Liu, Feng Gao, Jie Long, Hangyu Chen, Qianhui Zhu, Zilong He, Songnian Hu, Chuan He, Jian Lin, Yida Tang, Haibo Zhu

**Affiliations:** 1 State Key Laboratory for Bioactive Substances and Functions of Natural Medicines, Beijing Key Laboratory of New Drug Mechanisms and Pharmacological Evaluation Study, Institute of Materia Medica, Chinese Academy of Medical Sciences and Peking Union Medical College, Xian Nong Tan Street 1, Xicheng District, Beijing, 100050 China; 2 Department of Cardiology, Coronary Heart Disease Center, State Key Laboratory of Cardiovascular Disease, Fu Wai Hospital, National Center for Cardiovascular Diseases, Chinese Academy of Medical Sciences and Peking Union Medical College, 167 Beilishi Road, Beijing, 100037 China; 3 College of Chemistry and Molecular Engineering, Innovation Center for Genomics, Peking University, No. 5 Yiheyuan Road Haidian District, Beijing, 100871 China; 4 State Key Laboratory of Microbial Resources, Institute of Microbiology, Chinese Academy of Sciences, Beijing, China; 5 University of Chinese Academy of Sciences, Beijing, China; 6 Department of Chemistry, Department of Biochemistry and Molecular Biology, Howard Hughes Medical Institute, The University of Chicago, Chicago, IL 60637 USA

**Keywords:** 5-Hydroxymethylcytosine, Coronary artery disease, Epigenetic biomarkers

## Abstract

**Background:**

The 5-hydroxymethylcytosine (5hmC) DNA modification is an epigenetic marker involved in a range of biological processes. Its function has been studied extensively in tumors, neurodegenerative diseases, and atherosclerosis. Studies have reported that 5hmC modification is closely related to the phenotype transformation of vascular smooth muscle cells and endothelial dysfunction. However, its role in coronary artery disease (CAD) has not been fully studied.

**Results:**

To investigate whether 5hmC modification correlates with CAD pathogenesis and whether 5hmC can be used as a biomarker, we used a low-input whole-genome sequencing technology based on selective chemical capture (hmC-Seal) to firstly generate the 5hmC profiles in the circulating cell-free DNA(cfDNA) of CAD patients, including stable coronary artery disease (sCAD) patients and acute myocardial infarction (AMI) patients. We detected a significant difference of 5hmC enrichment in gene bodies from CAD patients compared with normal coronary artery (NCA) individuals. Our results showed that CAD patients can be well separated from NCA individuals by 5hmC markers. The prediction performance of the model established by differentially regulated 5hmc modified genes were superior to common clinical indicators for the diagnosis of CAD (AUC = 0.93) and sCAD (AUC = 0.93). Specially, we found that 5hmC markers in cfDNA showed prediction potential for AMI (AUC = 0.95), which was superior to that of cardiac troponin I, muscle/brain creatine kinase, and myoglobin.

**Conclusions:**

Our results suggest that 5hmC markers derived from cfDNA can serve as effective epigenetic biomarkers for minimally noninvasive diagnosis and prediction of CAD.

## Background

Coronary artery disease (CAD) remains a leading cause of mortality worldwide and was responsible for an estimated 8.14 million deaths (16.8%) in 2013 [1]. Clinical diagnosis of CAD is currently based on symptoms, electrocardiograms (ECGs), cardiac markers, stress testing, coronary computed tomographic angiography (CTA), and coronary angiography (CAG) are used for [2–4]; however, all these methods have limitations. Invasive CAG is regarded as the diagnostic “gold standard” [5]; however, specialized technology and high cost limit CAG to a selected population [2]. In addition, many individuals who undergo invasive CAG are found to have normal coronary arteries [6]. Symptom-based diagnosis might sometimes be inaccurate with episodes of myocardial ischemia or infarction occurring after atypical symptoms in some patients with CAD, especially in patients who are elderly or have diabetes [7]. In other noninvasive approaches to diagnose CAD, such as CTA, high sensitivity can only be achieved in the case of severe coronary stenosis, while early-stage atherosclerosis cannot be diagnosed. In addition, there are potential risks of radionuclide radiation-induced damage. The dependence on particular equipment and requirement for experience in interpreting the results also limits the large-scale use of these techniques.

As for cardiac markers, such as cardiac troponin I (cTnI), muscle/brain creatine kinase (CK-MB), and myoglobin (MYO), they are widely used for diagnosis and prediction of acute myocardial infarction (AMI) and also analyzed in this study. CTnI is considered as a crucial biomarker for diagnosis of myocardial damage [8, 9]. Indeed, elevated cTnI was also detected during reversible cellular injury while there is no myocardial necrosis [10]. The specificity and sensitivity of CK-MB are much lower than that of cTnI, and the interpretation of elevation of CK-MB alone is not reliable [11–13]. Myoglobin (MYO) is highly sensitive but not cardiospecific [11]. In addition, so far, there is no effective method that can be used to early warning of CAD. Thus, a method capable of diagnosis and prediction of CAD with high specificity and sensitivity is still highly desirable.

In recent years, increasing attention has been paid to 5-hydroxymethylcytosine (5hmC), an relative stable derivative produced in the demethylation process of 5-methylcytosine (5mC) mediated by ten-eleven translocation (TET) protein family. To allow obtaining the genome-wide 5hmC distribution and base-resolution analysis of 5hmC, two sensitive and selective approaches have been established by our group, including selective chemical labeling (hmC-Seal) technology and Tet-assisted bisulfite sequencing [14, 15]. Genome-wide mapping of 5hmC distributions and dynamics in various human tissues has shown that it is mainly enriched in gene bodies, promoters, and enhancers and has a potential role in gene regulation in mammalian development and cell differentiation [16, 17]. 5hmC modification has been implicated in a wide range of biological processes, including brain development [17], neurodegenerative diseases [18, 19], and cancers [20, 21]. There is a study have shown that 5hmC involves in cardiomyocyte heart development and hypertrophy in mouse [16]. Besides, accumulating evidence suggest that 5hmC and its TET2 enzyme, one member of the TET family, play an important role in atherosclerosis and are not only involved in the regulation of vascular smooth muscle cell phenotype, but also closely related to endothelial dysfunction and inflammatory immune response [22–26]. It was also found that 5hmC and TET2 were markedly absent in atherosclerotic plaque, and the level of deletion was positively correlated with the degree of injury [22]. 5hmC may play an important role in the pathological process of atherosclerosis.

Recently, considerable interest has focused on 5hmC modification in cell-free circulating DNA (cfDNA) because it may provide a liquid biopsy-based approach for noninvasive diagnosis and prediction of human diseases [27, 28]. cfDNA comprises fragments of genomic DNA (gDNA) contained in plasma, which are derived from various apoptotic and necrotic cells [28]. Recent studies have found that cardiomyocyte death can be detected by cfDNA in ST-elevation myocardial infarction and sepsis or septic shock [28]. Optimized chemical labeling detection methods based on hMe-Seal with high sensitivity and specificity have been established to capture 5hmC modification even at 1 ng of cfDNA [29–32]. Using these optimized procedures, previous studies have confirmed that the performance of 5hmC in cfDNA is comparable to which in gDNA in tissue biopsy samples for cancer diagnosis [29, 32]. Both can serve as biomarkers for cancer diagnosis, while the former may be used for minimally invasive diagnosis and prediction of human cancers. Since non-invasive biomarkers for CAD diagnosis and prediction are needed and 5hmC is involved in atherosclerosis, we investigate whether 5hmC modification correlates with CAD pathogenesis and whether 5hmC in cfDNA can be used as a biomarker.

Here, we employed hmC-Seal sequencing method for rapid, reliable, and precise sequencing of 5hmC in plasma cfDNA from 111 patients with CAD and 56 normal coronary artery (NCA) individuals. Our results demonstrated that CAD patients and NCA individuals had distinct differences in 5hmC enrichment. 5hmC markers derived from plasma cfDNA can be used to noninvasively diagnose of CAD, particularly used to predict AMI.

## Methods

### Participants and study design

Participants over 18 years old with complete information on medical history and clinical and biochemical parameters were recruited between October 2017 and March 2018 from Fuwai Hospital, the National Center for Cardiovascular Diseases of China. On the basis of clinical symptoms, signs, laboratory tests, ECG, and CAG results, which showed the extent of arterial blockage and myocardial injury, participants were divided into three groups. Patients with no plaques or stenosis in coronary arteries that included unexplained chest pain constituted the NCA group. Patients were considered eligible for the stable CAD (sCAD) group if coronary angiography showed ≥ 50% of the luminal diameter of at least one native coronary vessel. Patients hospitalized for myocardial infarction were excluded. The patients in the AMI group had ischemic chest pain and increased values of cardiac enzymes, with or without ST-T changes on the ECG. Patients admitted with chest pain and suspected of AMI were submitted to conventional ECG. They were also assessed using point-of-care testing, including cTnI, MYO, and CK-MB, 1, 3, and 6 h after admission to the emergency room. Blood samples were collected in EDTA. All enrolled participants in the NCA group, sCAD group, and AMI group who were suspected of CAD underwent CAG and had no history of unstable angina, myocardial infarction, stroke, cancers, or coronary revascularization. The angiographic data were confirmed independently by two observers in this study. Finally, 167 patients were enrolled, including 56 NCA individuals, 53 sCAD patients, and 58 AMI patients.

### Assessment of clinical and biochemical parameters

Height was measured to the nearest 0.1 cm using a tape rule, and weight was measured to the nearest 0.1 kg using calibrated platform scales. Body mass index was calculated as body weight (kg) divided by the square of height (m^2^). Smoking was defined as smoking for at least one cigarette per day for over a year. Alcohol consumption was defined as at least 20 g/day for men and 10 g/day for women for over a year. Blood pressure was measured using a mercury sphygmomanometer. Readings of systolic blood pressure and diastolic blood pressure were taken twice at a five-minute interval, during which the participants had rested on a chair. The average of these two readings was used for current analyses. Notably, an additional reading would be taken in the presence of an over 5 mmHg discrepancy between these two measurements. The average of these three readings was used for further analyses. Arterial hypertension was defined as a systolic pressure of ≥ 140 mmHg or a diastolic pressure of ≥ 90 mmHg. Other biochemical variables were measured at the central laboratory.

### Peripheral blood collection and preparation of cfDNA

Peripheral blood from patients and NCA individuals was collected for cfDNA preparation. Briefly, 8 ml of peripheral blood was collected into Cell-Free DNA Collection Tubes (Roche). Within 4 h, plasma was prepared by centrifuging twice at 1350×*g* for 12 min at 4 °C and 13,500×*g* for 12 min at 4 °C. cfDNA was extracted using the Quick-cfDNA Serum & Plasma Kit (ZYMO) and then stored at − 80 °C. The fragment size of all the cfDNA samples was verified by nucleic acid electrophoresis before library preparation.

### 5hmC library construction and high-throughput sequencing

5hmC libraries for all samples were constructed with high-efficiency hmC-Seal technology [14]. First, 1–10 ng cfDNA extracted from plasma was end-repaired, 3′-adenylated using the KAPA Hyper Prep Kit (KAPA Biosystems) and then ligated with the Illumina compatible adapters. The ligated cfDNA was added in a glucosylation reaction in 25 μl solution containing 50 mM HEPES buffer (pH 8.0), 25 mM MgCl_2_, 100 μM UDP-6-N3-Glc, and 1 μM β-glucosyltransferase (NEB) for 2 h at 37 °C. Next, 1 μl DBCO-PEG4-biotin (Click Chemistry Tools, 4.5 mM stock in DMSO) was directly added to the reaction mixture and incubated for 2 h at 37 °C. Then, the DNA was purified using the DNA Clean & Concentrator Kit (ZYMO). The purified DNA was incubated with 2.5 μl streptavidin beads (Life Technologies) in 1× buffer (5 mM Tris pH 7.5, 0.5 mM EDTA, 1 M NaCl, and 0.2% Tween 20) for 30 min. The beads were subsequently washed eight times for 5 min with 1× buffer. All binding and washing steps were performed at room temperature with gentle rotation. Then, the beads were resuspended in RNase-free water and amplified with 14–16 cycles of PCR amplification. The PCR products were purified using AMPure XP beads (Beckman) according to the manufacturer’s instructions. The concentration of libraries was measured with a Qubit 3.0 fluorometer (Life Technologies). Paired-end 39 bp high-throughput sequencing was performed on the NextSeq 500 platform.

### Mapping and differentially modified regions detection

All sequencing raw data were trimmed using trim_galore (version 0.6.0) [33]. Adaptor sequences and low-quality sequences at the end of the sequences (quality score < 30, Q30) were trimmed off, and only the reads with a length greater than 20 bp were retained (parameters used: --paired --quality 30 --length 20). The remaining paired-end reads were mapped to the human genome (version hg19) using Bowtie 2 (version 2.1.0) [34], and then filtered with SAMtools (version 1.9) (parameters used: samtools view -f 2 -F 1548 -q 30) [35]. Only reads with Mapping Quality Score (MAPQ) > 30 were retained for the subsequent analysis. Then, samples sequencing depth with greater than 100× or unique mapping rate greater than 80% were selected. FeatureCounts of Subread version v1.5.3 was used to to count for overlap with genomic features [36]. Then, 5hmC-enriched regions (hMRs) were identified with MACS2 based on Poisson algorithm [37]. Genomic annotations of hMRs were performed by HOMER (version v4.10) [38]. All paired-end reads were converted into the bedgraph format normalized by bam2bedgraph (version 1.0.4) [39] and the genome wide distribution of 5hmC was visualized using the Integrated Genomics Viewer (IGV) (version 2.5.3) [40, 41].The metagene profile was generated using ngsplot (version 2.61). The 5hmC fragments per kilobase of transcript per million mapped reads (FPKM) of hMRs was calculated using the fragment counts in each hMR region obtained by bedtools [42].

### Detection of differential genes and functional enrichment analysis

After filtering out genes in chromosomes X and Y, differential modified genes in the autosomes between samples from CAD (sCAD + AMI) patients and NCA individuals were identified using DESeq2 (v1.24.0) package in R (version 3.6.0) [43, 44]. The differential modified 5hmC regions (differentially 5hmC enriched regions, DhMRs) in each comparison of two groups were obtained respectively with the criterion log_2_foldchange > 1 and *P* value < 0.05. Among them, CAD group contained sCAD and AMI group was compared with NCA group, sCAD group was compared with AMI group, NCA group was compared with sCAD group or AMI group. Unsupervised hierarchical clustering and heatmap analysis were performed by Pheatmap (version 1.8.0) in R package. Principal component analysis (PCA) was performed for the analysis of DhMRs using prcomp function in R package, with 80% confidence interval drawing core region. Functional and pathway enrichment analysis of differential 5hmc modified genes were performed by KOBAS (version 3.0) [45], which is a web server for gene/protein functional annotation mainly based on hypergeometric test. Subsequently, top 10 KEGG pathways or top 10 GO terms associated with human cardiovascular function were selected to display. The findMotifsGenome.pl of HOMER (version 4.11) was performed to find the corresponding binding proteins targeted to DhMRs of each two group comparison (e.g., NCA vs CAD; sCAD vs AMI, etc.). And motif information was obtained from the Homer motif database internally. For the result of motif enrichment in DhMRs, according to the enriched *P* value and the percentage of target sequences enriched with the binding motif which indicated transcription factor, top enriched known transcription factor binding motifs were shown which followed the approach of Zhang et al. [46].

### Feature selection and classifier construction

The Boruta (version 6.0.0) package in R was used to select the important 5hmC features in all detected DhMRs based on the random forest classifier, and then the randomForest package (version 4.6-14) in R was used to construct the classification model for 100 times [47]. The prediction effect of the model was evaluated by the area under the receiver operating characteristic curve (AUC), and the optimal score threshold is selected by the ROCR package in R to calculate the corresponding specificity and sensitivity [48]. The training and validation datasets of all differential genes were selected randomly with the proportion of 7:3. In other words, in each case, the model was trained on 70% of the data and results refer to the remaining 30% of the data which was used to test the performance of the model. The out-of-bag (OOB) error was used to optimize the parameter and evaluate the stability of the model. To further select the most reliable hydroxymethylation marker genes, both mean decrease accuracy (MDA) and the significance (*P* value) of two-tailed *t* tests were used to filter top candidate genes to show the classification capabilities of 5hmC marks which followed the approach of Zhang et al [46]. Briefly, the MDA of each gene which showed feature importance and contribution to the model was calculated internally by the model, and high MDA values referring to greater importance. Subsequently, the criterion with MDA > 2 and the *P* value < 0.01 of two-tailed *t* tests which were calculated by *t* test of R (version 3.6.) were leveraged to filter top potential gene marks.

### Statistical analysis

All continuous variables are presented as mean ± SD, and analysis of variance was used to compare means across four groups. Noncontinuous and categorical variables are presented as frequencies or percentages and were compared by using the χ^2^ test. A two-sided *P* value of < 0.05 was considered to indicate statistical significance. Statistical analysis was performed using SPSS version 23.0 (IBM Corp. Released 2016. IBM SPSS Statistics for Mac, Version 23.0. Armonk, NY, USA).

## Results

### Genome-wide 5hmC profiles of cfDNA differ among sCAD, AMI, and NCA groups

We firstly used a low-input whole-genome sequencing technology based on hmC-Seal technology [14] to generate the 5hmC profile in cfDNA of CAD patients. Baseline characteristics and laboratory data are shown in Table 1. Among the three groups, significant differences were detected in Gender (*p* = 0.014), age (*p* = 0.012), drinking (*p* = 0.004), smoking (*p* < 0.0001), hypertension (< 0.0001), diabetes mellitus (< 0.0001), hyperlipemia (< 0.0001), systolic blood pressure (*p* = 0.003), hemoglobin (*p* < 0.0001), fasting blood glucose (*p* < 0.0001), hemoglobin A1c (*p* = 0.019), serum creatinine (*p* < 0.0001), lactate dehydrogenase (*p* < 0.0001), aspartate transaminase (*p* < 0.0001), uric acid (*p* = 0.032), total cholesterol (TC) (*p* = 0.029), high-sensitive C-reactive protein (*p* < 0.0001), creatine kinase (*p* < 0.0001), CK-MB (*p* < 0.0001), cTnI (*p* < 0.0001), and MYO (*p* < 0.0001). No significant difference was found in Body mass index (*p* = 0.889), high-density lipoprotein cholesterol (*p* = 0.482), low-density lipoprotein cholesterol (LDL-C) (*p* = 0.093), and triglycerides (0.635). According to the unique mapping rate, there were good sequencing quality observed among the all samples and no apparent difference observed among the three groups (Additional file 2: Figure S1A and S1B).

**Table 1:** Baseline characteristics

	Total (*n* = 167)	NCA (*n* = 56)	sCAD (*n* = 53)	AMI (*n* = 58)	*P* value
Demographic characteristics					
Age	57.76 ± 11.80	54.23 ± 12.5	58.30 ± 11.41	60.67 ± 10.73	0.012
Gender/Male (%, M)	125 (74.9%)	35 (62.5%)	40 (75.5%)	50 (86.2%)	0.014
Clinical characteristics					
BMI, kg/m^2^	25.18 ± 3.19	25.22 ± 2.70	25.01 ± 3.05	25.30 ± 3.76	0.889
Smoking (%, Y)	95 (56.9%)	17 (30.4%)	34 (64.2%)	44 (75.9%)	< 0.0001
Drinking (%, Y)	72 (43.1%)	15 (26.8%)	31 (58.5%)	26 (44.8%)	0.004
Diabetes mellitus (%, Y)	46 (27.5%)	4 (7.1%)	21 (39.6%)	21 (36.2%)	< 0.0001
Hyperlipemia (%, Y)	101 (60.5%)	20 (35.7%)	41 (77.4%)	40 (69.0%)	< 0.0001
Laboratory characteristics					
Hemoglobin, g/L	134.68 ± 18.37	142.31 ± 16.84	134.26 ± 19.38	127.68 ± 16.11	< 0.0001
FBG, mmol/L	6.58 ± 2.61	5.35 ± 1.04	7.04 ± 2.60	7.34 ± 3.21	< 0.0001
HbA1c, %	6.30 ± 1.29	5.91 ± 0.87	6.55 ± 1.51	6.44 ± 1.34	0.019
Scr, μmol/L	83.59 ± 17.90	75.72 ± 16.22	87.21 ± 18.87	87.87 ± 16.23	< 0.0001
Uric acid, μmol/L	331.47 ± 86.72	324.13 ± 79.45	356.62 ± 86.68	315.58 ± 89.76	0.032
LDH, IU/L	276.64 ± 237.57	171.68 ± 37.18	188.83 ± 44.50	458.24 ± 331.19	< 0.0001
hsCRP, mg/L	3.86 ± 5.12	1.94 ± 2.41	2.78 ± 3.57	6.69 ± 6.81	< 0.0001
ALT, IU/L	35.04 ± 32.59	30.09 ± 30.37	35.64 ± 38.92	39.28 ± 27.91	0.320
AST, IU/L	55.25 ± 80.02	23.29 ± 13.74	25.998 ± 16.53	112.95 ± 114.11	< 0.0001
HDL-C, mmol/L	1.22 ± 1.15	1.19 ± 0.31	1.10 ± 0.35	1.36 ± 1.90	0.482
LDL-C, mmol/L	2.56 ± 0.97	2.79 ± 0.87	2.48 ± 1.09	2.42 ± 0.91	0.093
TC, mmol/L	4.29 ± 1.16	4.62 ± 0.92	4.07 ± 1.37	4.17 ± 1.09	0.029
TG, mmol/L	2.12 ± 5.98	1.83 ± 1.19	1.76 ± 0.88	2.73 ± 10.08	0.635
NT-proBNP, pg/mL	496.40 ± 1504.93	86.25 ± 221.68	224.41 ± 396.73	1140.94 ± 2397.50	< 0.0001
CK, IU/L	372.70 ± 659.52	99.80 ± 57.03	118.38 ± 134.82	868.59 ± 929.25	< 0.0001
cTnI, ng/mL	8.64 ± 20.38	0.15 ± 0.30	0.89 ± 2.94	23.92 ± 28.94	< 0.0001
MYO, ng/mL	256.08 ± 572.39	28.26 ± 11.63	48.63 ± 47.83	665.59 ± 830.84	< 0.0001
CK-MB, ng/mL	34.05 ± 65.07	3.42 ± 4.95	7.06 ± 21.94	88.30 ± 85.29	< 0.0001

Data are means ± SD or number (percentage) of subjects. *BMI* body mass index, *FBG* fasting blood glucose, *HbA1c* hemoglobin A1c, *Scr* serum creatinine, *LDH* lactate dehydrogenase, *hsCRP* hypersensitive C-reactive protein, *ALT* alanine aminotransferase, *AST* aspartate transaminase, *HDL*-*C* high-density lipoprotein cholesterol, *LDL*-*C* low-density lipoprotein cholesterol, *TC* total cholesterol, *TG* triglycerides, *NT*-*proBNP* N-terminal pro-brain natriuretic peptide, *CK* creatine kinase, *cTnI* cardiac troponin I, *MYO* myoglobin, *CK*-*MB* MB isoenzyme of creatine kinase, *NCA* normal coronary artery, *sCAD* stable coronary artery disease, *AMI* acute myocardial infarction. The chi-squared test was used for comparison of categorical variables and one-way analysis of variance (one-way ANOVA) was used for continuous variables. The P value<0.05 was regarded as statistically significant

**Fig. 1:**
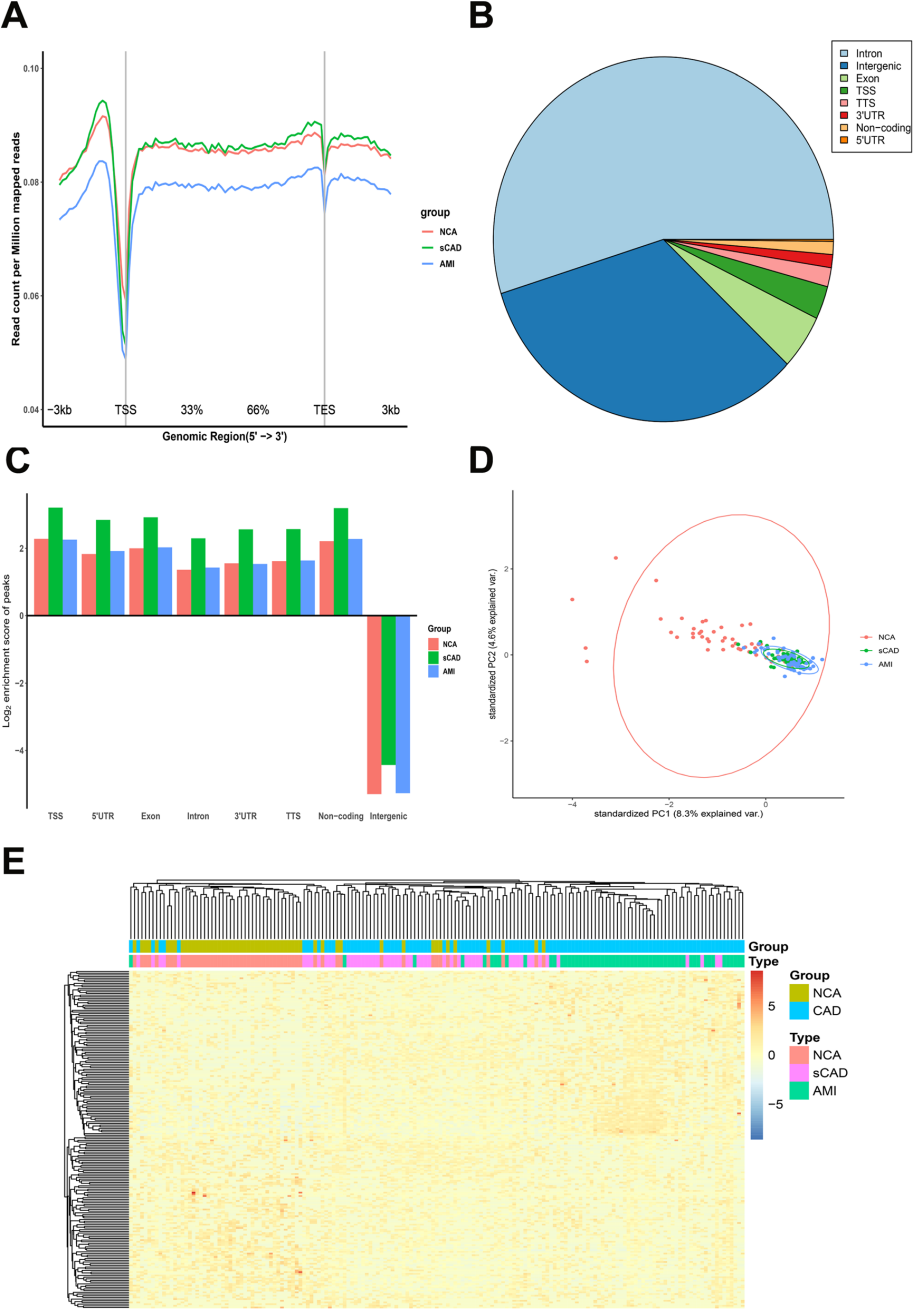
Genome-wide distribution of 5hmC in plasma samples of sCAD, AMI patients, and NCA individuals. **a** Metagene profiles of cell free 5hmC in NCA, sCAD and AMI samples. **b** The pie chart shows the overall genomic distribution of hMRs in cfDNA. **c N**ormalized enrichment score of hMRs across distinct genomic regions relative to that expected in NCA, sCAD, and AMI samples, with positive values indicating enriched more than expected. **d** Principle component analysis (PCA) plot of 5hmC FPKM in cfDNA from CAD (AMI + sCAD) and NCA samples. **e** Heatmap of the top 200 potential 5hmC markers in CAD and NCA groups. Unsupervised hierarchical clustering was performed across genes and samples. *CAD* coronary artery disease, *NCA* normal coronary artery, *AMI* acute myocardial infarction, *sCAD* stable coronary artery disease, *TSS* transcription start site, *TTS* transcription termination site, *FPKM* fragments per kilobase of transcript per million mapped reads

To ask whether or not the genome-wide 5hmC profiles of plasma cfDNA had difference in sCAD, AMI, and NCA groups, we first compared the distribution of 5hmC along the gene bodies of the three groups and found that the overall normalized read density of cfDNA 5hmC were significantly different (Fig. 1a). AMI group showed the lowest 5hmC level in gene bodies among the three groups. And there was a comparable 5hmC level in sCAD and NCA group. Then, we analyzed 5hmC enrichment in different genomic characteristic regions by HOMER [38] and the overall genomic distribution of 5hmC-enriched regions (hMRs) in all samples were showed in Fig. 1b. The genome-wide analysis of hMRs of NCA, sCAD, and AMI groups showed that hMRs were mostly enriched in transcription start site (TSS) and non-coding region in gene bodies, whereas fewer hMRs were found in intergenic regions (Fig. 1c), which was consistent with previous studies showing that the majority of 5hmC in mammals is enriched in the intragenic and promoter regions and correlated with gene expression [31, 46].

Next, to further explore the 5hmC signal changes among coronary artery diseases, we identified the differentially regulated 5hmc modified genes (genes with differential 5hmC levels) in all CAD patients (sCAD patients + AMI patients) and NCA individuals by DESeq2 package (*P* value < 0.05, log2foldchange > 1). We detected 170 upregulated 5hmc modified genes and 421 downregulated 5hmc modified genes based on the fragments per kilobase of transcript per million mapped reads (FPKM) of each gene in the all CAD group compared with the NCA group (Additional file 2: Figure S1C). Total upregulated and downregulated 5hmC gene id for each group were presented a supplementary excel sheet (Additional file 1: Table S1).

The results suggested that cfDNA 5hmC profiles of NCA individuals, sCAD, and AMI patients indeed displayed significant differences. To evaluate the classification effects of 5hmC signals for NCA, sCAD, and AMI samples, we carried out the principal component analysis (PCA) for genes with differentially regulated 5hmC levels and found that CAD samples (sCAD samples + AMI samples) showed prominent signatures and could be readily separated from NCA samples (Fig. 1d). However, there were few different signatures showed in sCAD and AMI samples and they could not be separated from each other (Fig. 1d). Then, we clustered the top 200 differentially regulated 5hmc modified genes (100 up and 100 down) detected from all CAD patients and NCA individuals by unsupervised hierarchical clustering method. Similarly, the results showed that the majority of CAD samples were well separated from NCA samples; meanwhile, AMI, sCADs, and NCA samples just tended to differentiate from each other (Fig. 1e). Thus, the above results meant that differentially regulated 5hmc modified genes may have the potential to distinguish CAD patients from non-CAD patients.

### 5hmC markers derived from cfDNA can be used to separated CAD patients from non-CAD patients

**Fig. 2:**
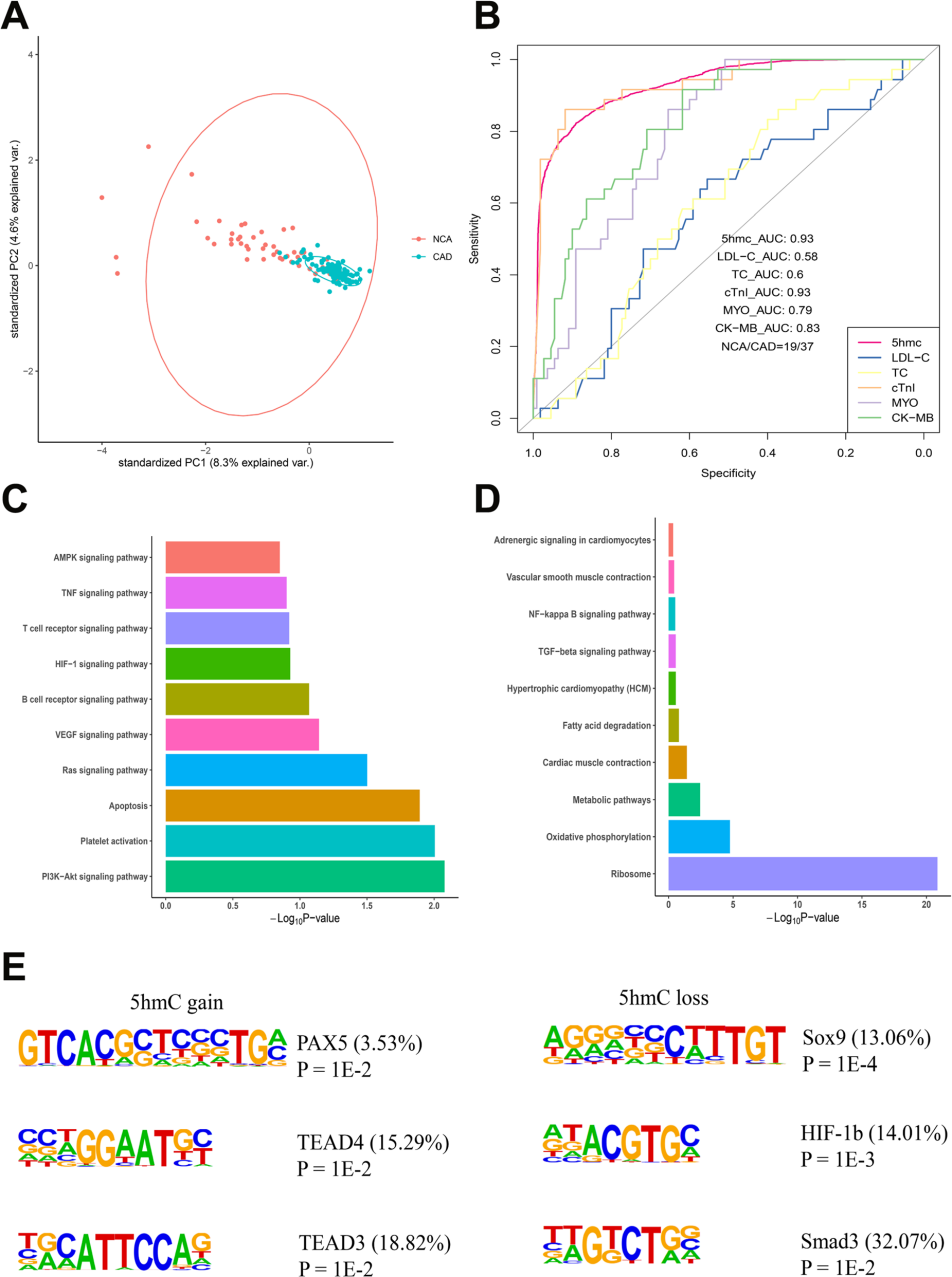
Performance of potential 5hmC markers for identification of CAD patients and non-CAD patients. **a** Principle component analysis (PCA) plot of 5hmC FPKM in cfDNA from CAD and NCA samples. **b** Receiver operating characteristic (ROC) curve of the classification model with potential 5hmC markers and clinical indicators, including LDL-C, TC, cTnI, MYO, and CK-MB in the validating set (19 NCA vs. 37 CAD samples). The true positive rate (sensitivity) is plotted in function of the false positive rate (specificity). **c** KEGG functional enrichment analysis of genes with significant 5hmC increase in CAD samples. **d** KEGG functional enrichment analysis of genes with significant 5hmC decrease in CAD samples. **e** Top enriched known transcription factor binding motifs detected in DhMRs in CAD and NCA groups (left: 5hmC gain; right: 5hmC loss). Motif information was obtained from the Homer motif database. The value in parenthesis represents the percentage of target sequences enriched with the binding motif of the indicated transcription factor. *CAD* coronary artery disease, *NCA* normal coronary artery, *TSS* transcription start site, *TTS* transcription termination site, *FPKM* fragments per kilobase of transcript per million mapped reads, *AUC* area under the curve, *TC* total cholesterol, *LDL*-*C* low-density lipoprotein cholesterol, *cTnI* cardiac troponin I, *MYO* myoglobin, *CK*-*MB* MB isoenzyme of creatine kinase, *DhMRs* differentially hydroxy methylated regions, *PAX5* paired box protein Pax-5, *TEAD4* transcriptional enhancer factor TEF-3, transcriptional enhancer factor TEF-5, *SOX9* SRY-type box 9, *HIF*-*1b* hypoxia-inducible factor 1 beta, *Smad3* mothers against decapentaplegic homolog 3

We found that the average profile of the 5hmC level showed obvious 5hmC loss in the CAD group (Additional file 2: Figure S1D). Thus, we speculated 5hmC characteristics detected in cfDNA can be utilized for CAD classification. The PCA analysis result preliminarily demonstrated that CAD samples could be readily separated from NCA samples by genes with differentially regulated 5hmC levels (Fig. 2a). To evaluate the classification effects of 5hmC signals for CAD and non-CAD samples, we extracted the top 30 mean decrease Gini (MDG) differentially 5hmC markers by Boruta to construct a classification model based random forest classifier. With the tree numbers of the model increasing, out-of-bag (OOB) error rates decreased and tended to be stable at ~ 500 (Additional file 2: Figure S1E). According to the result, the prediction performance of the model achieved 82% sensitivity and 89% specificity (AUC = 0.93) for patient classification in validating set (19 NCA individuals vs. 37 CAD patients, Fig. 2b). Then, we compared the performance of the model with that of clinical cardiovascular risk factors and indicators, including TC, LDL-C, cTnI, CK-MB, and MYO. We chose 5.98 mmol/L, 1.8 mmol/L, 0.08 ng/mL, 6 ng/mL, and 70 ng/mL, respectively, as the cutoff points for TC, LDL-C, cTnI, CK-MB, and MYO, according to the testing standards of Fuwai Hospital. The AUC values of TC, LDL-C, cTnI, CK-MB, and MYO were 0.6, 0.58, 0.93, 0.83, and 0.79, indicating lower sensitivity and specificity than that of 5hmC classification model (AUC = 0.93), except for cTnI (Fig. 2b). The results suggested that cTnI did have a good diagnostic performance for coronary heart disease, and differentially 5hmC markers also showed a diagnostic performance comparable to that of cTnI.

Next, we used both MDG (MDG > 2) and the significance (*P* value) of two-tailed *t* tests (*P* value < 0.01) to filter the top 30 genes to find out the most reliable 5hmC marker genes. There were six genes that satisfy this condition (Additional file 2: Figure S1F). Besides, we performed KEGG functional enrichment analysis to study the biological significance of differentially 5hmC markers. We found that genes with upregulated 5hmC signal were mainly distributed in CAD-related pathways, such as PI3K-Akt signaling pathway, Platelet activation, apoptosis, Ras signaling pathway, AMPK signaling pathway (Fig. 2c). Genes with decreased 5hmC signal were enriched in several CAD-related pathways including metabolic pathways, cardiac muscle contraction, fatty acid degradation, and NF-kappa B signaling pathway (Fig. 2d).

Finally, motif enrichment analysis in DhMRs was performed to display the correlation of 5hmC changes with potential interactions of binding proteins. Our results showed that the motif of transcriptional enhancer factor TEF-3 (TEAD4) was significantly enriched in 5hmC gain regions (*P* ≤ 0.01) (Fig. 2e), which was a transcriptional factor regulating gene expression in muscle and to control cell proliferation and associated with coronary artery disease risk [49]. On the contrary, the motif of SRY-type box 9 (SOX9) was significantly enriched in 5hmC loss regions (*P* = 1E-4) (Fig. 2e). SOX9 was a common transcriptional regulator of a large portion of the heart valve development related and fibrosis-related genes, which was activated under conditions of ischemic injury and was considered to be a potential therapeutic target for cardiac fibrosis [50, 51]. Thus, our results indicated that CAD patients could be readily separated from NCA individuals by differentially regulated 5hmc-modified genes. CAD patients and NCA individuals showed apparent differences in both 5hmC enrichment and potentially interacting binding proteins.

### 5hmC markers from plasma cfDNA distinguish sCAD patients from NCA individuals with high specificity and high sensitivity

**Fig. 3:**
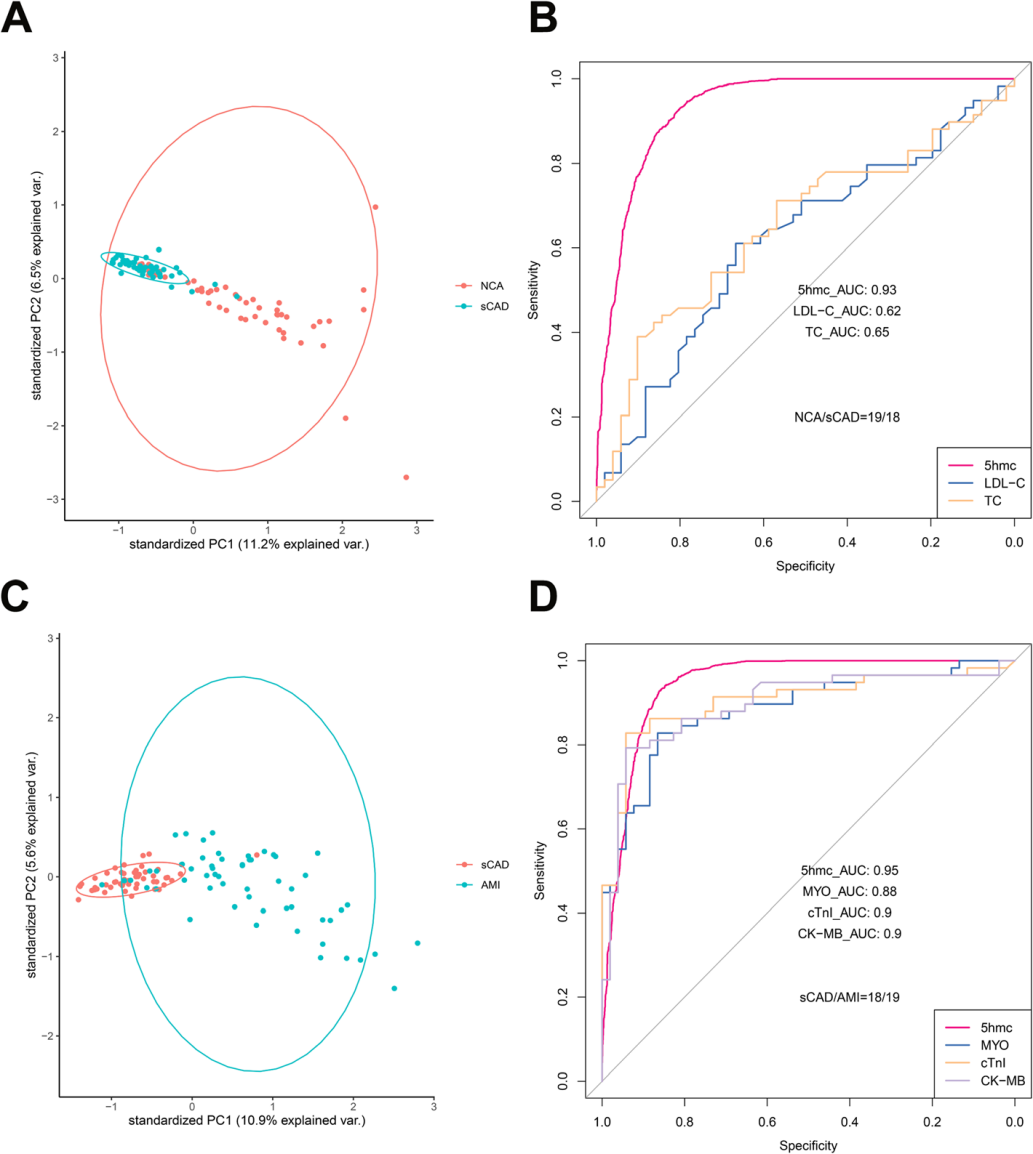
Performance of potential 5hmC markers for diagnosis and prediction of stable coronary artery disease and acute myocardial infarction. **a** Principle component analysis (PCA) plot of 5hmC FPKM in cfDNA from sCAD and NCA samples. **b** Receiver operating characteristic (ROC) curve of the classification model with potential 5hmC markers and clinical indicators, including TC and LDL-C in the validating set (18 sCAD patients vs. 19 NCA individuals). The true positive rate (sensitivity) is plotted in function of the false positive rate (specificity). **c** Principle component analysis (PCA) plot of 5hmC FPKM in cfDNA from AMI and sCAD samples. **d** Receiver operating characteristic (ROC) curve of the classification model with potential 5hmC markers and clinical indicators, including cTnI, MYO and CK-MB in the validating set (19 AMI patients vs. 18 sCAD patients). The true positive rate (sensitivity) is plotted in function of the false positive rate (specificity). *NCA* normal coronary artery, *sCAD* stable coronary artery disease, *AMI* acute myocardial infarction, *FPKM* fragments per kilobase of transcript per million mapped reads, *AUC* area under the curve, *TC* total cholesterol, *LDL*-*C* low-density lipoprotein cholesterol, *cTnI* cardiac troponin I, *MYO* myoglobin, *CK*-*MB* MB isoenzyme of creatine kinase

Although there was no significant difference in 5hmC level between the sCAD group and NCA groups, we detected 85 upregulated 5hmc modified genes and 804 downregulated 5hmc modified genes in sCAD group compared with NCA group (Additional file 2: Figure S1C). In addition, the PCA analysis indicated that differential 5hmC markers could distinctly separate these two groups (Fig. 3a). To further evaluate the performance of these differentially 5hmC markers in distinguishing sCAD samples from NCA samples, we then also extracted the top 30 MDG differentially 5hmC markers to construct a classification model. With the tree numbers of the model increasing, OOB error rates decreased accordingly and tended to be stable at ~ 600 (Additional file 3: Figure S2A). The prediction performance of the model reached an AUC value of 0.93, with a sensitivity of 93% sensitivity, and a specificity of 80% specificity for sCAD patient classification in validating set (18 sCAD patients vs. 19 NCA individuals) (Fig. 3b). Then, we compared the diagnostic performance of differentially 5hmC markers with that of TC and LDL-C in discriminating NCA from sCAD using plasma samples. The AUC values of TC and LDL-C were 0.65 and 0.62 (Fig. 3b), respectively, indicating much lower performance than that of differentially 5hmC markers in cfDNA (AUC = 0.93). These results suggested that differentially 5hmC markers in cfDNA may be effective epigenetic markers for minimally noninvasive diagnosis of sCAD.

In addition, six genes of the above 30 genes satisfied both MDG > 2 and the two-tailed *t* tests *P* value < 0.01 (Additional file 3: Figure S2B). KEGG functional enrichment analysis showed that genes with significant 5hmC gain or loss in the sCAD group were mainly enriched in Ras signaling pathway, Chemokine signaling pathway, AMPK signaling pathway, vascular smooth muscle contraction, VEGF signaling pathway, apoptosis, HIF-1 signaling pathway, and platelet activation, which are closely associated with the occurrence and development of sCAD (Additional file 3: Figure S2C and S2D).

### 5hmC markers from plasma cfDNA show prediction potential for acute myocardial infarction superior to that of cTnI, CK-MB, and MYO

The enrichment level of 5hmC in cfDNA was significantly different between sCAD group and AMI group. Therefore, we speculated that 5hmC features may have the potential to differentiate these two groups. We carried out the PCA analysis and found that AMI samples showed obvious signatures and could be readily separated from sCAD samples (Fig. 3c). Then, we sought to estimate the performance of differentially 5hmC markers for patient classification. We also utilized the top 30 MDG differentially 5hmC markers to construct a classification model based random forest algorithm. With the tree numbers of the model increasing, OOB error rates decreased and tended to be stable at ~ 600 (Additional file 4: Figure S3A). The prediction performance of the model achieved a sensitivity of 93% and a specificity of 86% (AUC = 0.95) in the validating set (19 AMI patients vs. 18 sCAD patients), which was superior to that of cTnI (AUC = 0.90), MYO (AUC = 0.88), and CK-MB (AUC = 0.90) (Fig. 3d).

In addition, we found five potential gene were both MDG > 2 and the two-tailed *t* tests *P* value < 0.01 (Additional file 4: Figure S3B). To further investigate whether the potential marker genes are associated with the occurrence of AMI, we performed KEGG functional enrichment analysis. The results showed that genes with significant 5hmC gain or loss in the AMI group were mainly enriched in apoptosis, vascular smooth muscle contraction, VEGF signaling pathway, platelet activation, HIF-1 signaling pathway, cardiac muscle contraction, and metabolic pathways, which are closely associated with acute myocardial infarction (Additional file 4: Figure S3C and S3D).

## Discussion

5hmC, as a novel epigenetic biomarker, plays a critical role in gene expression regulation and involves in various biological processes, including tumors, cardiovascular, neurological diseases, and metabolic diseases [52]. Although the signal of cfDNA 5hmC in the blood is low, it has the potential to be biomarkers in different cancer types [53]. More recently, a diagnostic model based on features from cfDNA 5hmC in the blood showed the potential for early detection of hepatocellular carcinoma [54]. So the signal features of cfDNA 5hmC in the blood could be reliable biomarkers for different diseases. In this study, we utilized hmC-Seal sequencing method to detected cfDNA 5hmC of CAD patients to, so as to try to uncover reliable biomarkers for CAD.

First, we found that CAD patients and NCA individuals had prominent differences in 5hmC enrichment in plasma cfDNA (Figs. 1a, d and 2a). Second, our results showed that CAD patients can be well separated from non-CAD patients by 5hmC markers derived from cfDNA (Fig. 2b). The prediction performance of the model established by 5hmC markers were superior to TC, LDL-C, CK-MB, and MYO for the diagnosis of CAD (Fig. 2b). Third, 5hmC markers derived from cfDNA can use to diagnose sCAD with high sensitivity and specificity (Fig. 3b). In addition, we found that 5hmC markers derived from cfDNA could pre-warning the occurrence of AMI and the prediction potential was superior to that of cTnI, CK-MB, and MYO (Fig. 3d). Furthermore, we found that 5hmC markers mainly distributed in pathways which were highly correlated with the pathogenesis of CAD (Fig. 2c, d). The potentially interacting binding proteins targeted to differentially modified 5hmC regions played an important role in CAD (Fig. 2e). Taken together, these findings indicated that 5hmC markers derived from cfDNA can serve as effective epigenetic biomarkers for minimally noninvasive diagnosis for CAD and prediction of AMI.

The occurrence of AMI is very fatal. If the risk of AMI can be predicted, early intervention may significantly improve the prognosis of patients. However, there is still no effective method to alert its occurrence. In our study, we firstly found that 5hmC markers derived from cfDNA could pre-warning the occurrence of AMI and the prediction potential was superior to that of cTnI, CK-MB and MYO. If these findings are supported by further expanded studies, it may offer promising prediction strategies for AMI.

Previous studies have showed that 5hmC is enriched in contractile VSMCs but reduced in dedifferentiated VSMCs and improves endothelial cell function via upregulation of autophagy [22, 55, 56]. Recent study analyzing 5hmC on a genome-wide scale in cardiomyocytes has shown that 5hmC modification plays an important role in myocardial pathophysiology [16]. In our study, we also found that 5hmC markers in plasma cfDNA were enriched in various CAD-related signaling pathways and the potential interacting binding proteins targeted to differential modified 5hmC regions played an important role in CAD. For example, SOX9 is a regulator of a large portion of the fibrosis-related genes that become activated under conditions of ischemic injury and associated with CAD [50, 51]. RUNX2 is closely associated with calcification of vascular smooth muscle cells [35]. Interestingly, we found that most genes of the top 30 MDG differentially 5hmC markers were pseudogenes. The experimental data obtained during recent years indicate this understanding of the nature of pseudogenes is not entirely correct, and many pseudogenes are functionally significant elements of the genome and may play a regulatory role in the form of non-coding RNA [57–59]. Thus, we speculated that 5hmC regulated gene expression by regulating the production of pseudogene RNA.

Nevertheless, our research still has some limitations. One of the limitations of our research is that we do not know the source of the cfDNA. Cell-free DNA (cfDNA) in the circulating blood originates from dying cells from different tissues, which release DNA into the peripheral bloodstream upon degradation after cell death [60]. A recent study has shown, beyond blood cells, that cfDNA is derived from vascular endothelial cells, hepatocytes, and other cells in healthy individuals [61]. So it could reflect multi-organ processes in the body. Secondly, the sample size of our research is still relatively small. Recently, 5hmC-Seq has been applied in clinical research and shown the potential for diagnostic and prognostic in different disease [53]. In addition, it has shown high sensitivity and specificity in the early detection of gastrointestinal tumors and liver cancer compared with the clinical gold standard [54]. Thus, 5hmC has emerged to be a novel class of cancer epigenetic biomarkers with promise in precision medicine. Therefore, the next step we should do is to increase the amount of samples to find more efficient biomarkers of 5hmC in CAD. Thirdly, many factors, like age, smoking, drinking, diabetes, etc., may affect the enrichment of 5hmC and may need further perform stratification and correlation analysis of key factors. Besides, since it was a cross-sectional study, this study could not observe in prospective way and thus could not identify the causal effect. Study duration should be extended to perform longitudinal studies, which will be more convinced to confirm the relationship between 5hmC and CAD. Finally, the regulatory mechanism of 5hmC and these CAD-related genes and pathways is still unclear. We speculate that the mechanism may be related to the regulation of downstream transcripts or the chromatin spatial structure [62]. Thus, prospective studies are really required for further study.

## Conclusions

Our results suggest that 5hmC markers derived from cfDNA can serve as effective epigenetic biomarkers for minimally noninvasive diagnosis and prediction of CAD, and show prediction potential for acute myocardial infarction superior to that of cTnI, CK-MB, and MYO.

## Supplementary information

**Additional file 1: Table S1.** Total upregulated and downregulated 5hmC gene id for each group. The *P* value<0.05 and log2foldchange>1 was regarded as statistically significant.

**Additional file 2: Figure S1.** Quality of raw sequencing data within samples of the three groups. a Unique mapping rate of the samples. Each boxplot represents all the samples of each group. b Unique mapping reads numbers for the groups. Each boxplot represents all the samples of each group.c Differentially regulated 5hmC genes detected in cfDNA from the three groups. dMetagene profiles of cell free 5hmC in CAD and NCA samples. e Out-of-bag (OOB) error rates in CAD and NCA groups by different trees Random-Forest built. f Scatterplot showing the MDG and the significance of two-tailed t-tests for the top 30 potential markers in the CAD and NCA groups. Red dots refer to significant differential genes.

**Additional file 3: Figure S2.** Pathways and genes with 5hmC variations between sCAD and NCA groups. a Out-of-bag (OOB) error rates in sCAD and NCA groups by different trees Random-Forest built. b Scatterplot showing the MDG and the significance of two-tailed t-tests for the top 30 potential markers in the sCAD and NCA groups. Red dots refer to significant differential genes. c KEGG functional enrichment analysis of genes with significant 5hmC increase in sCAD samples. d KEGG functional enrichment analysis of genes with significant 5hmC decrease in sCAD samples.

**Additional file 4: Figure S3.** Pathways and genes with 5hmC variations between sCAD and AMI groups. a Out-of-bag (OOB) error rates in sCAD and AMI groups by different trees Random-Forest built. b Scatterplot showing the MDG and the significance of two-tailed t-tests for the top 30 potential markers in the sCAD and AMI groups. Red dots refer to significant c KEGG functional enrichment analysis of genes with significant 5hmC increase in AMI samples. d KEGG functional enrichment analysis of genes with significant 5hmC decrease in AMI samples.

## Data Availability

The datasets supporting the conclusions of this article are included within the article and its additional files. All other datasets used and analyzed during the current study are available from the corresponding author on reasonable request.

## Abbreviations

5hmC: 5-Hydroxymethylcytosine
5mC: 5-Methylcytosine
AMI: Acute myocardial infarction
AUC: Area under ROC curves
CAD: Coronary artery disease
CAG: Coronary angiography
cfDNA: Cell-free DNA
CK-MB: Muscle/brain creatine kinase
CTA: Tomographic angiography
cTnI: Cardiac troponin I
ECG: Electrocardiograms
FPKM: Fragments per kilobase of transcript per million mapped reads
hMRs: 5hmC-enriched regions
IGV: Integrated Genomics Viewer
LDL-C: Low-density lipoprotein cholesterol
MYO: Myoglobin
NCA: Normal coronary artery
OOB: Out-of-bag
PCA: Principal component analysis
ROC: Receiver operating characteristic
sCAD: Stable CAD
TC: Total cholesterol
TET: Ten-eleven translocation
