# Supplementary figures and images for: 5-Hydroxymethylcytosine signatures in circulating cell-free DNA as diagnostic and predictive biomarkers for coronary artery disease
Source: Clin Epigenetics. 2020 Jan 21;12:17. doi: 10.1186/s13148-020-0810-2 (PMC6974971; doi:10.1186/s13148-020-0810-2)

**A**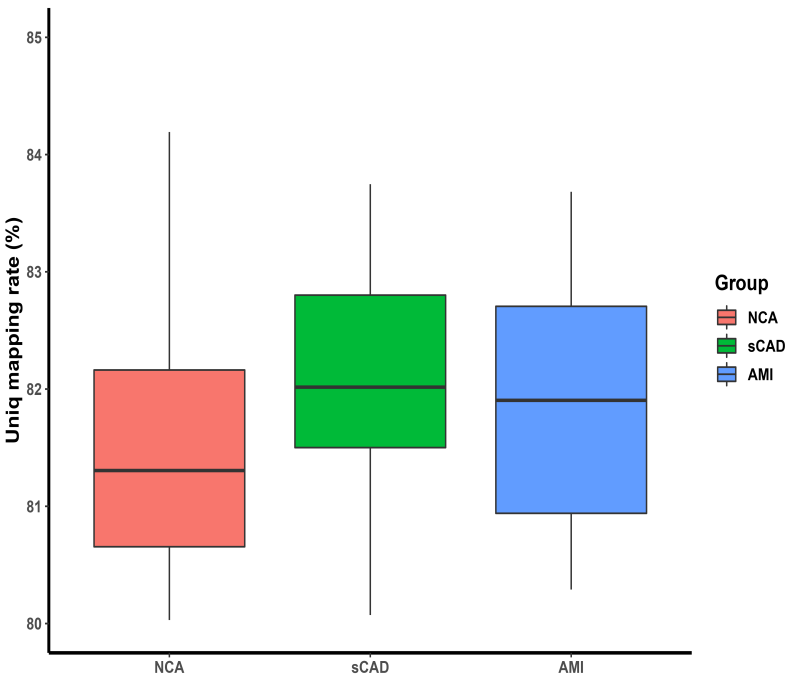**B**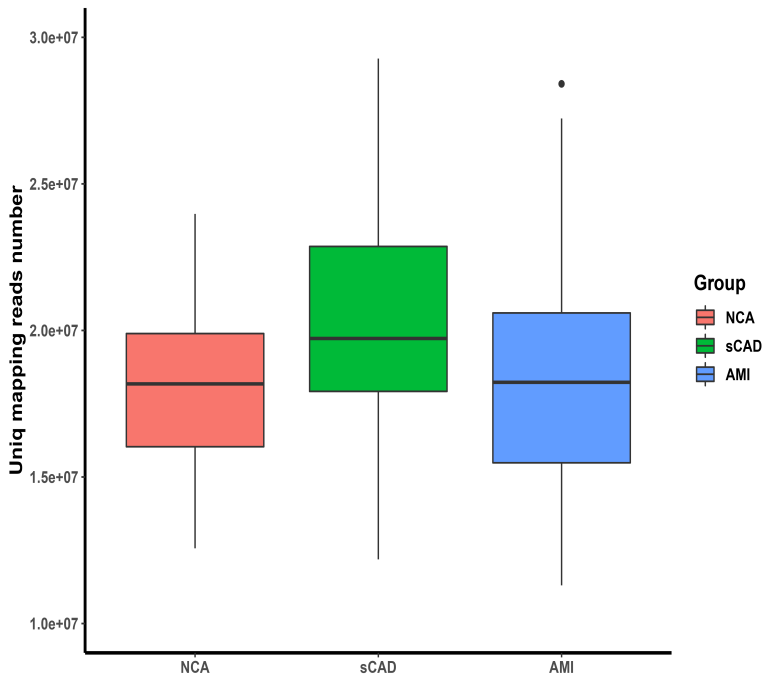**C**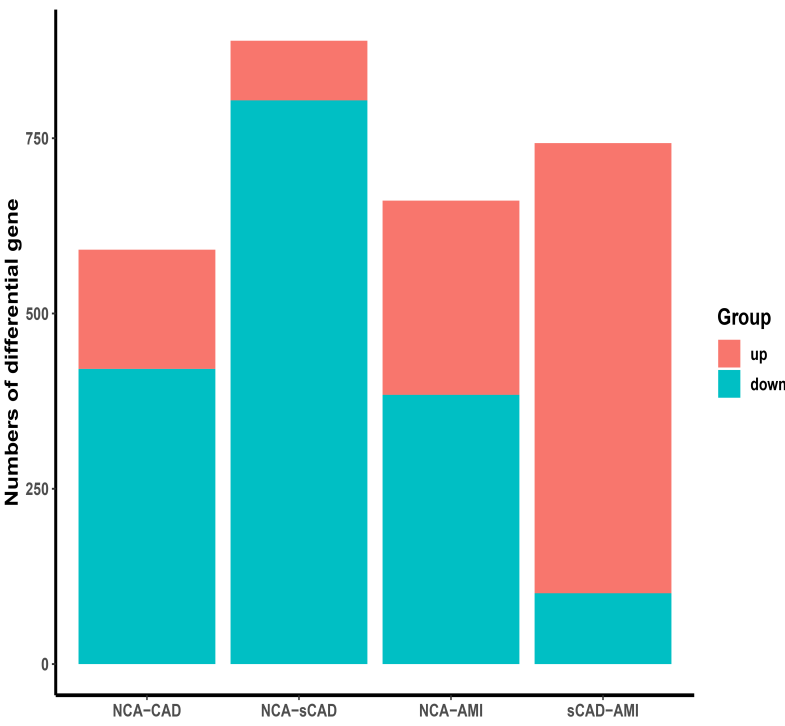**D**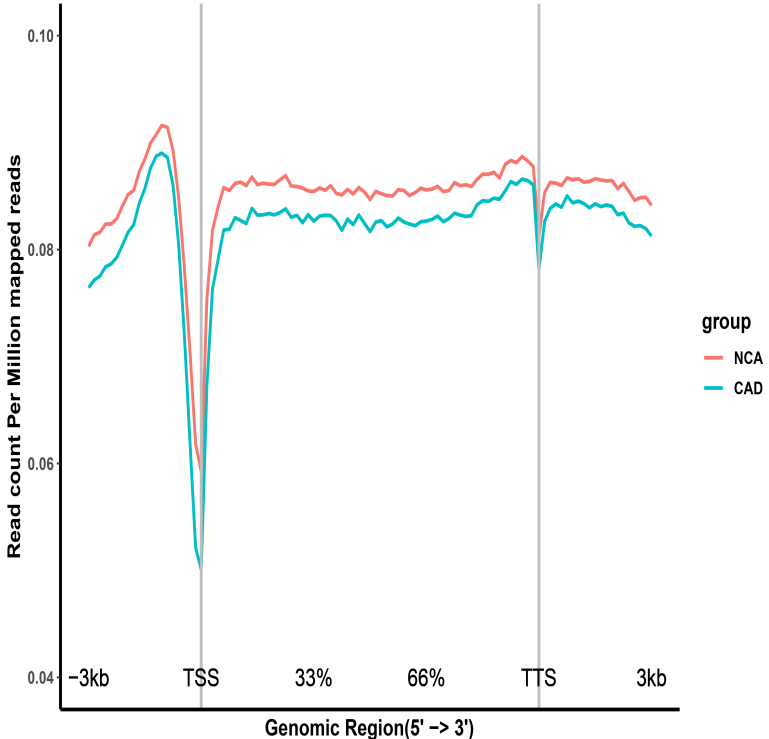**E**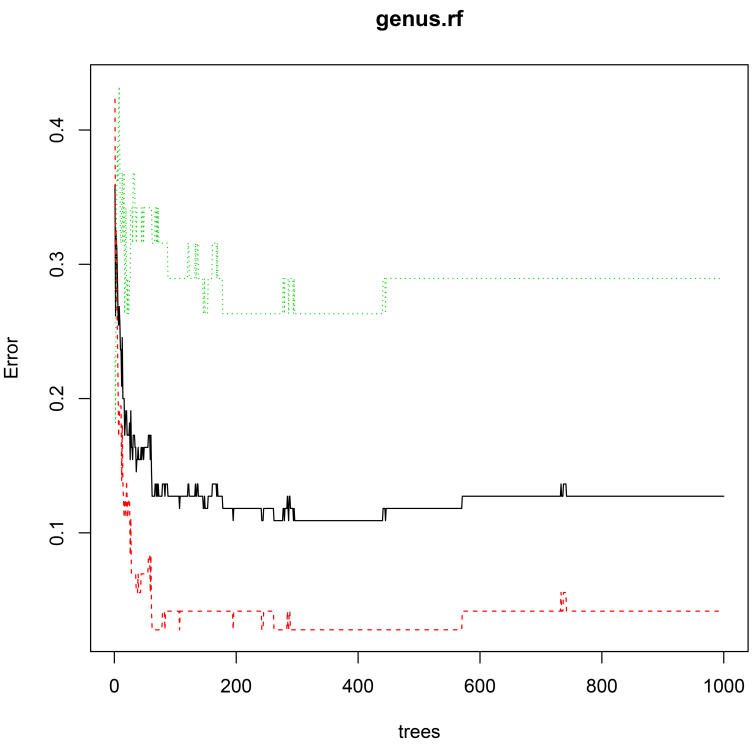**F**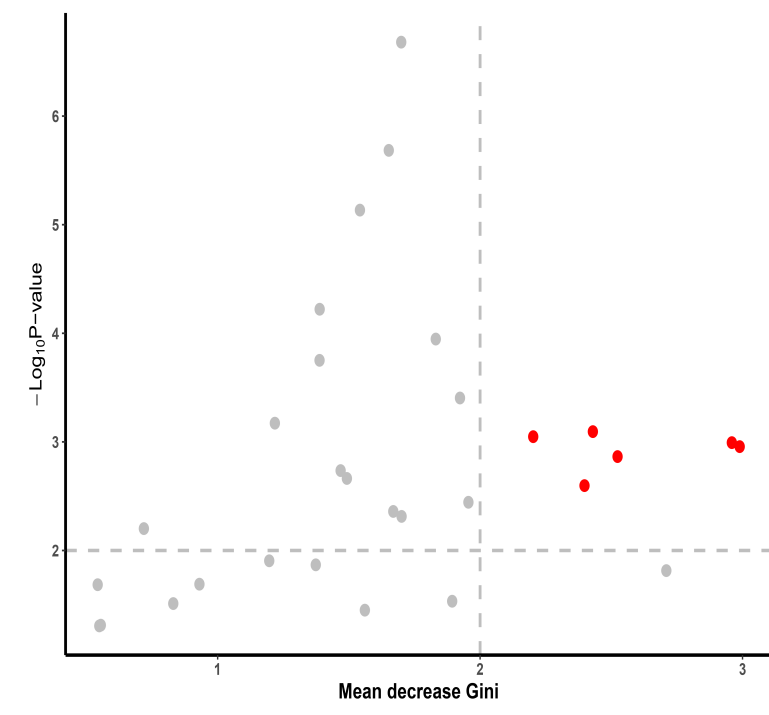

Supplement: Supplementary file 2 — Additional file 2: Figure S1. Quality of raw sequencing data within samples of the three groups. a Unique mapping rate of the samples. Each boxplot represents all the samples of each group. b Unique mapping reads numbers for the groups. Each boxplot represents all the samples of each group.c Differentially regulated 5hmC genes detected in cfDNA from the three groups. dMetagene profiles of cell free 5hmC in CAD and NCA samples. e Out-of-bag (OOB) error rates in CAD and NCA groups by different trees Random-Forest built. f Scatterplot showing the MDG and the significance of two-tailed t-tests for the top 30 potential markers in the CAD and NCA groups. Red dots refer to significant differential genes. [file 13148_2020_810_MOESM2_ESM.pdf]

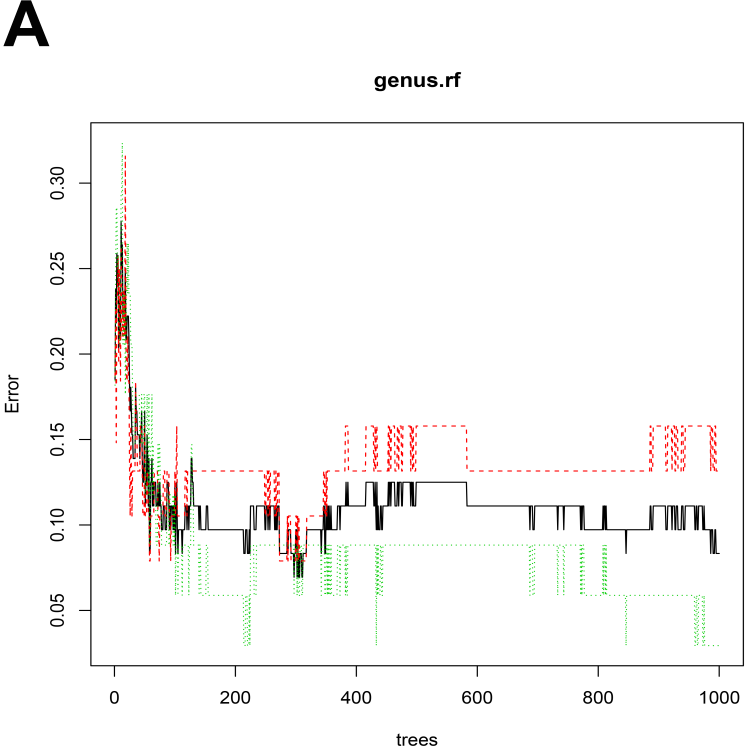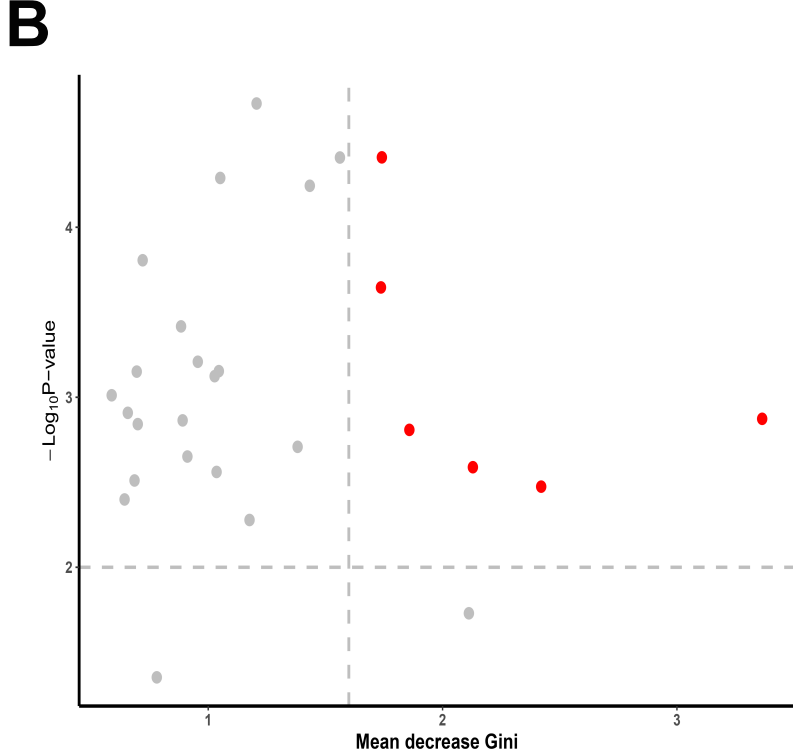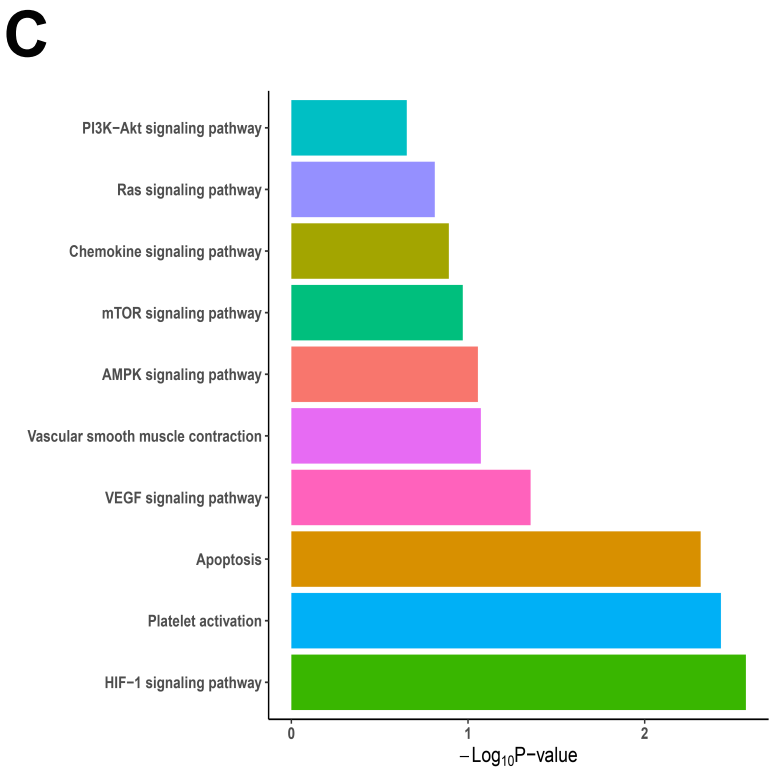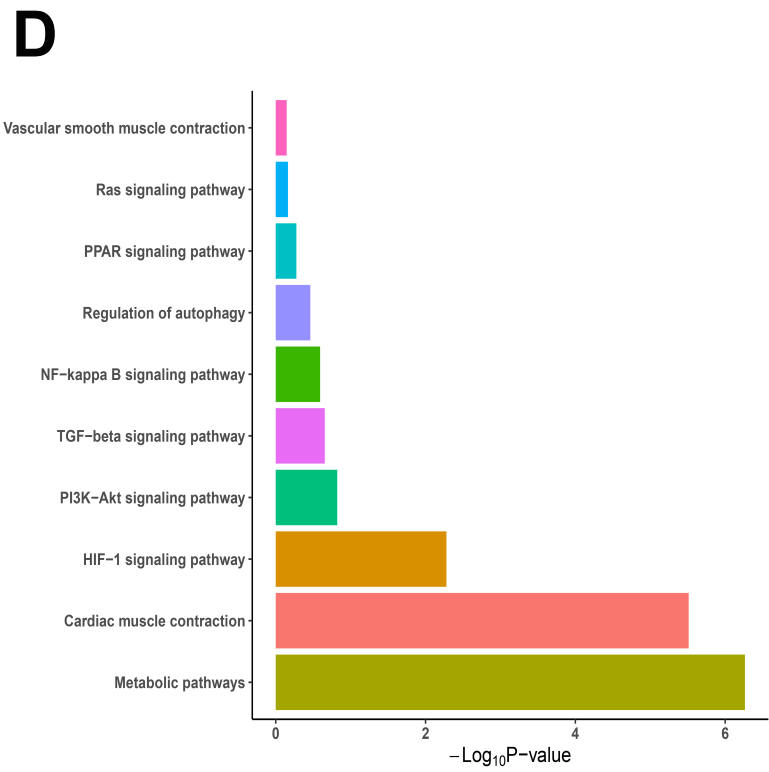

Supplement: Supplementary file 3 — Additional file 3: Figure S2. Pathways and genes with 5hmC variations between sCAD and NCA groups. a Out-of-bag (OOB) error rates in sCAD and NCA groups by different trees Random-Forest built. b Scatterplot showing the MDG and the significance of two-tailed t-tests for the top 30 potential markers in the sCAD and NCA groups. Red dots refer to significant differential genes. c KEGG functional enrichment analysis of genes with significant 5hmC increase in sCAD samples. d KEGG functional enrichment analysis of genes with significant 5hmC decrease in sCAD samples. [file 13148_2020_810_MOESM3_ESM.pdf]

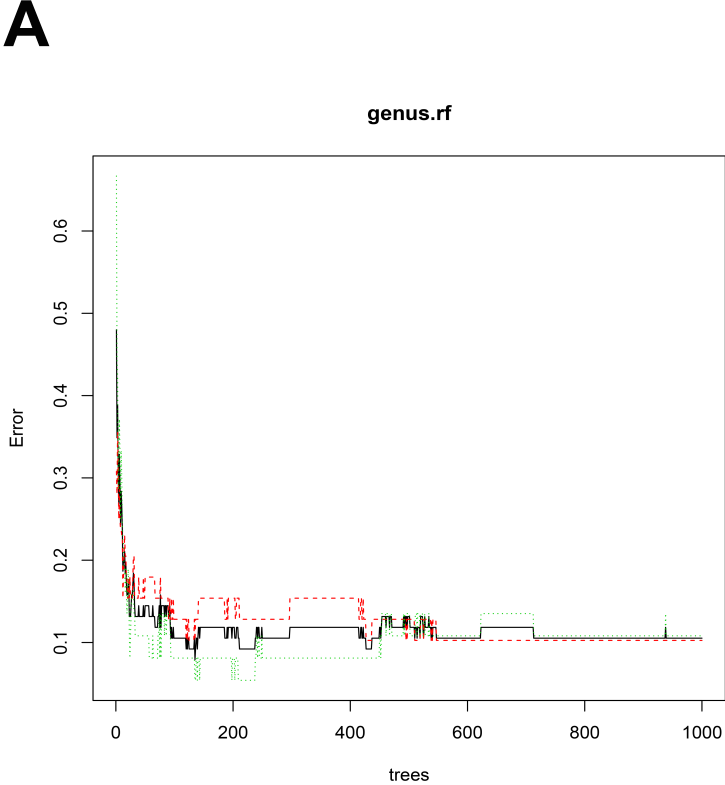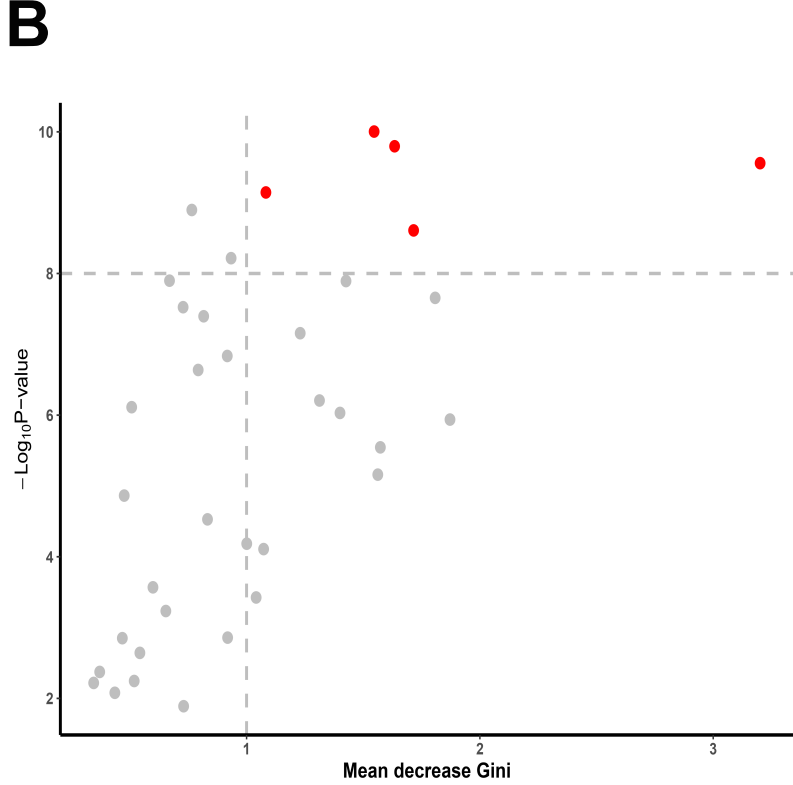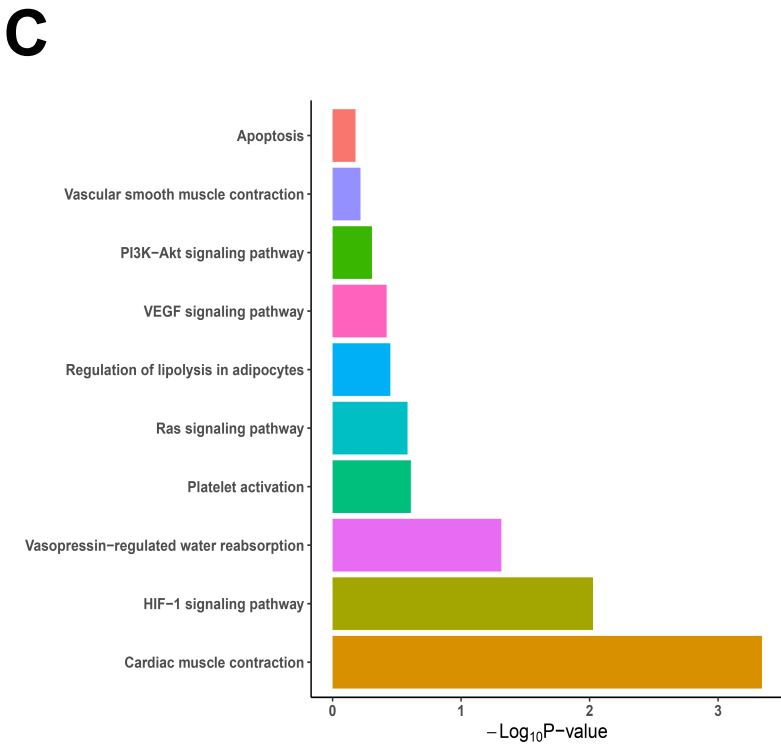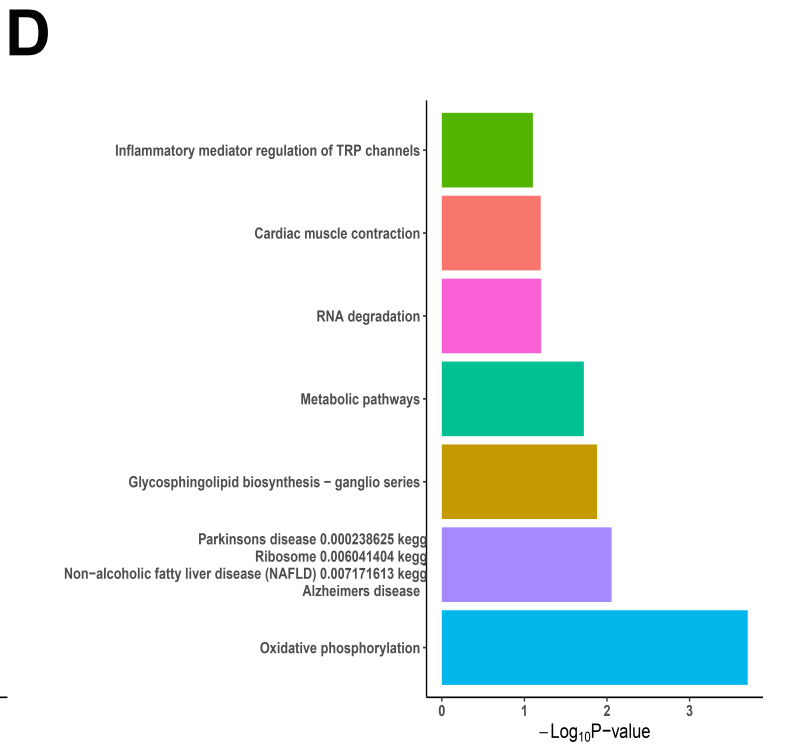

Supplement: Supplementary file 4 — Additional file 4: Figure S3. Pathways and genes with 5hmC variations between sCAD and AMI groups. a Out-of-bag (OOB) error rates in sCAD and AMI groups by different trees Random-Forest built. b Scatterplot showing the MDG and the significance of two-tailed t-tests for the top 30 potential markers in the sCAD and AMI groups. Red dots refer to significant c KEGG functional enrichment analysis of genes with significant 5hmC increase in AMI samples. d KEGG functional enrichment analysis of genes with significant 5hmC decrease in AMI samples. [file 13148_2020_810_MOESM4_ESM.pdf]
